# A GeXP-Based Assay for Simultaneous Detection of Multiple Viruses in Hospitalized Children with Community Acquired Pneumonia

**DOI:** 10.1371/journal.pone.0162411

**Published:** 2016-09-14

**Authors:** Le Wang, Mengchuan Zhao, Zhongren Shi, Zhishan Feng, Weiwei Guo, Shuo Yang, Lanping Liu, Guixia Li

**Affiliations:** 1 Institute of Pediatric Research, Children’s Hospital of Hebei Province, Shijiazhuang 050031, China; 2 Graduate School of Hebei Medical University, Shijiazhuang 050000, China; 3 Department of Laboratory Medicine, Children’s Hospital of Hebei Province, Shijiazhuang 050031, China; Kliniken der Stadt Köln gGmbH, GERMANY

## Abstract

The GeXP-based assay has recently been developed for simultaneous detection of multiple pathogens. So far, the application of the GeXP assay to test larger clinical samples has hardly been reported. Community-acquired pneumonia (CAP) is the leading cause of death in children worldwide and a substantial proportion of childhood CAP is caused by viruses. Rapid and accurate diagnosis of virus infection is important for the clinical management of CAP. In this study, we explored the GeXP assay for simultaneous detection of 20 types/subtypes of viruses in hospitalized children with CAP. A total of 1699 nasopharyngeal swabs were prospectively collected and viral nucleic acid was extracted and assayed. Using viral genomic DNA or RNA as template, we showed that at the concentration of 10^4^ copies of DNA or RNA of each virus/μl, all 20 target viruses were simultaneously identified by the GeXP assay. Fifteen control microorganisms, in contrast, failed to be amplified by the assay. About 65% of cases tested in this study had viral infection, with patients aged <3 years having a 70% positive rate, significantly higher than that in patients aged > 3 years (40%). The most frequently detected virus was RSV followed by PIV3, HRV, ADV and HBoV. Seasonal distribution analysis revealed that RSV was the most predominant in autumn and winter, while in spring and summer PIV3 and RSV were the most frequently identified with similar positive percentages. One hundred twenty randomly-chosen samples tested by the GeXP assay were re-evaluated by mono-RT-PCR, the results showed 97.5% diagnosis agreement between these 2 methods. Our findings suggest that the GeXP assay could be a valuable diagnostic tool for virus infection in pediatric patients with CAP.

## Introduction

Community acquired pneumonia (CAP) in children can be potentially serious and often results in hospitalization [1–3]. It is the leading cause of respiratory morbidity and mortality in children worldwide [4–6]. Etiologic agents of CAP include bacteria and virus [6–12]. While bacterial infection appears to occur mostly in older children, viral infection is more prevalent in patients under the age of 5 years [9, 12]. Viruses commonly detected in children with CAP include respiratory syncytial virus (RSV), influenza A and B (Flu A and B), parainfluenza viruses (PIV), adenovirus (ADV), human rhinovirus (HRV), and human metapneumovirus (HPMV) etc. [7, 9–12].

Multiplex reverse transcription polymerase chain reaction (multiplex RT-PCR) combined with automated capillary electrophoresis has provided a platform for multi-target genome analysis. An example of this technology is the GenomeLab GeXP Genetic Analysis System (https://www.beckmancoulter.com/wsrportal/bibliography?docname=BR-11776A.pdf) developed by Beckman Coulter (Crea, CA, USA). The principle of the GeXP multiplex amplification assay (the GeXP-based assay) is that diverse genomic sequences are specifically amplified in one reaction, and the amplicons with different sizes labelled with fluorescence dye are then separated and distinguished by automated capillary electrophoresis. For multiplex PCR, two sets of primers are used: one set of forward and reverse primers that are chimeric (target primers) containing a target sequence and a universal tag at the 5’-end. The other set of forward and reverse primers (universal primers) targeting the tag is fluorescence labeled. With the use of a significantly higher concentration of the universal primers than target primers in the reaction system, the target sequence is amplified with the chimeric primers in first few cycles, and then the amplification is overtaken by the universal primers, eventually generating amplicons containing target sequence and fluorescence-labeled tag [13]. Using the GeXP-based assay, Hu et al. was able to simultaneously genotype nine serotypes of enteroviruses [13]; Zhang et al. detected and differentiated 11 duck viruses in one reaction [14]; and Li et al simultaneously detected multiple human respiratory viruses in nasopharyngeal aspirates from patients with pneumonitis or bronchopneumonia [15]. So far, the application of the GeXP assay to test larger clinical samples has rarely been reported. In this study, we used the GeXP-based assay for simultaneous detection of 20 types/subtypes of viruses in 1699 nasopharyngeal specimens collected from hospitalized children with CAP.

## Material and Methods

### Experimental design

Sensitivity and specificity of the GeXP assay were determined using genomic nucleic acid (RNA or DNA) from various pathogens as template. Afterwards, nasopharyngeal swab samples from 1699 patients with CAP were tested using the GeXP assay. In view of commonly identified and newly emerged respiratory viruses [7, 9–12, 16], 20 types/subtypes of viruses were selected as the detection targets. The target panel contained following viruses: Flu A, Flu B, influenza H5N1 (H5N1), PIV-1, -2, and -3, RSV, HRV, ADV, HMPV, human bocavirus (HBoV), coronavirus HKU1/OC43 (HCoV-HKU1/OC43), coronavirus NL63/229E (HCoV-NL63/229E), SARS-associated coronavirus (SARS), influenza A (H1N1) pdm09 (swH1N1), influenza N2 (N2, nomenclature of this strain is based on the neuraminidase gene, GenBank access number: J02156.1), influenza N1 (N1), seasonal H1N1 (SeH1N1), influenza H1 (H1) and influenza H3 (H3). Fifteen other microorganisms were chosen as control. The control panel consisted of cytomegalovirus, *Ureaplasma urealyticum*, Epstein-Barr virus, *Staphylococcus aureus*, *Staphylococcus epidermidis*, *Streptococcus pyogenes*, *Streptococcus pneumoniae*, *Enterococcus faecalis*, *Pseudomonas aeruginosa*, *Klebsiellar pneumonia*, *Escherichia coli*, measles virus, mumps virus, rubella virus and *Mycobacterium tuberculosis*. All genomic nucleic acid in the target and control panels was obtained from Health Gene Technologies Co., Ltd. (Ningbo, China) and quantified using spectrophotometry. Plasmid pcDNA3.1 (+) (Thermo Fisher Scientific Co Ltd., Shanghai, China) was used as internal control. A reverse transcription Kit and a PCR Kit used for multiplex RT-PCR were obtained from Health Gene Technologies Co., Ltd.

### Primer design

Alignment of the conserved genomic regions was performed and a pair of chimeric primers was designed for each target virus using the Primer Premier Software version 6.0 (Premier Biosoft, Palo Alto, CA). Each primer contained a gene-specific sequence with a universal tag at the 5’-end. A pair of primers targeting the universal tag was used with the forward primer labeled with cy5 fluorescent dye. Information of all primers was listed in Table 1. All primers were synthesized by Sangon Biotech (Shanghai, China).

**Table 1:** Information of all primers.

Target			Sequence 5'-3'	Amplicon size(bp)
1	FluA	F	GTACGACTCACTATAGGGATGGACAAAKCGTCTACGCTGC	140
		R	AGGTGACACTATAGAATAGACAAGACCAATTCTGTCACCT	
2	ADV	F	GTACGACTCACTATAGGGAGCTGGACATGACYTTYGAGGT	144
		R	AGGTGACACTATAGAATAGATGACGCCGCGGTGYGGCT	
3	PIV1	F	GTACGACTCACTATAGGGATGATGAATACGCATATATTG	152
		R	AGGTGACACTATAGAATACAATATCTCATTATTACCYGG	
4	HBoV	F	GTACGACTCACTATAGGGATTGGAGAAATCACTGCTACTG	156
		R	AGGTGACACTATAGAATAGAAATGAGTTTGGAATTTTA	
5	HRV	F	GTACGACTCACTATAGGGAGTAGCACACGSGGCTCTT	162
		R	AGGTGACACTATAGAATACCTGGCAGATGAGGCWG	
6	229E	F	GTACGACTCACTATAGGGATTCTTTTAGAACATCACAATTTTGTTCATC	182
		R	AGGTGACACTATAGAATACTGAGCATGATTTCTTTACTTGGAAAG	
7	SARS	F	GTACGACTCACTATAGGGACTTAGCTACTTCRTTGCTTCY	199
		R	AGGTGACACTATAGAATAGAATGATCACAGCACCAATGA	
8	N2	F	GTACGACTCACTATAGGGAGCACAGTAGTAATGACTGATG	202
		R	AGGTGACACTATAGAATACAGACACAYCTGACAYCAGG	
9	FluB	F	GTACGACTCACTATAGGGACTGAAATGGTTCGAGCATTATAG	207
		R	AGGTGACACTATAGAATAAAGCTGGCAGAARAGYTGCA	
10	PIV3	F	GTACGACTCACTATAGGGAAGAAGGAAGATTACTTYTACT	213
		R	AGGTGACACTATAGAATACCCATGGACATTCRTTGTTTC	
11	H5N1	F	GTACGACTCACTATAGGGAGGCWATAGATGGAGTCACC	217
		R	AGGTGACACTATAGAATACATGAGAACCAGAAGTTCAGCATT	
12	PIV2	F	GTACGACTCACTATAGGGAAACCATTTACCTAAGTGATGG	225
		R	AGGTGACACTATAGAATAGATATGAATCTTTCAATAAAGG	
13	HMPV	F	GTACGACTCACTATAGGGATGCCTCTTAAGAGAYGAYCARG	235
		R	AGGTGACACTATAGAATATGTGCTAACTTTGCAYGGGTARTT	
14	swH1N1	F	GTACGACTCACTATAGGGATTGAGCTCAGTRTCATCWTTTGAA	239
		R	AGGTGACACTATAGAATATCCCTTTATCATTAATGTAGGATTTGC	
15	RSV	F	GTACGACTCACTATAGGGAATTACCAAGTGARGTAARTCTCTGCA	246
		R	AGGTGACACTATAGAATAATACATAATCACACCCGTTAGARAA	
16	SeH1N1	F	GTACGACTCACTATAGGGATATGCTTTTGCARTGAYTAGAMG	253
		R	AGGTGACACTATAGAATAAAGGGATATTCCTTAYTCCTGTAAMC	
17	H1	F	GTACGACTCACTATAGGGAGCYGAYCARAAGAGCACACAYAAT	260
		R	AGGTGACACTATAGAATACCAAAGTRCTTTCATYTTCCAYT	
18	H3	F	GTACGACTCACTATAGGGAGCTGGTTCAGAGTTCCTCAACA	277
		R	AGGTGACACTATAGAATAAAACTCCAGTKTGCCKGATGA	
19	OC43	F	GTACGACTCACTATAGGGAATCCCAWTGACAATCRAASGG	290
		R	AGGTGACACTATAGAATAGAATGTTGCTAAGTAYACTCAYTTA	
20	Internal Control	F	GTACGACTCACTATAGGGAGCCAGATATACGCGTTGACA	322
		R	AGGTGACACTATAGAATAGGGCGTACTTGGCATATGAT	
21	N1	F	GTACGACTCACTATAGGGAGGRGCCTTGYTYAATGRCA	330
		R	AGGTGACACTATAGAATAACACATGCACATTCAGAYTCTYG	

The underlined sequences were the universal Tag sequences.

### Sensitivity analysis

To evaluate the sensitivity of the GeXP assay, nucleic acid from all 20 target viruses and the internal control pcDNA3.1 (+) DNA were mixed to make the template pool. The target pool was serially diluted (10-fold) to obtain dilutions containing 10^4^−10^1^ copies of DNA or RNA of each virus/μL. For multiplex reverse transcription (RT), the mix of all reverse primers (RT primer) from 20 target viruses (Table 1) was used. RT was performed in a total volume of 10 μL containing 1 μL of template pool dilution, 2 μL 5× RT buffer, 1 μL RT primer mix (1 pmol each primer), 0.5 μL (20 units) reverse transcriptase, 1 μL RNase inhibitor (4 units) and 4.5 μL nuclease-free water. RT was carried out as follows, 48°C for 1 min; and then 42°C for 60 min. The reaction was terminated by incubation at 95°C for 5 min. For multiplex PCR, the mix of all target virus primers including both forward and reverse, and the mix of universal primers including both forward and reverse were used. Multiplex PCR was performed as follows, 3.0 μL RT product was mixed with 1 μL 10×PCR Buffer, 2 μL MgCl_2_ (25 mM), 1 μL viral primers mix (1.25 pmol each primer), 1 μL universal primer mix (12.5 pmol each primer), 1 μL Solution X and 1.0 μL Taq DNA Polymerase. The PCR was completed on a thermocycler (Veriti Thermal Cycler, Applied Biosystems China, Beijing, China) in following steps: step 1, 94°C for 1 min; step 2, 94°C for 30 s, 60°C for 30 s and 70°C for 30 s. Step 2 was repeated for 40 cycles followed by incubation at 70°C for 1 min. The amplified products were then assessed using the GenomeLab GeXP Genetic Analysis System (Beckman Coulter).

### Specificity analysis

To evaluate the specificity of the GeXP assay, nucleic acid from all 15 control microbes and pcDNA3.1 (+) DNA were mixed to make the control pool. The control pool containing 1.0 ng of DNA or RNA from each microbe/μL was prepared and 1 μL was used for multiplex RT-PCR analysis as described above.

### Analysis of multiplex RT-PCR products with the automated GenomeLab GeXP Genetic Analysis System

Multiplex RT-PCR products were analyzed with the GenomeLab GeXP Genetic Analysis System as follow, 220 μL of Separation Buffer was added into the separation plate, and 38.5 μL Loading Solution, 0.5 mL DNA size standard-400 and 1μL multiplex PCR product were added into the reader plate covered with a drop of mineral oil. The analysis was then performed in an automated manner following the established protocol and the data were compiled by the GeXP system software provided by Beckman Coulter. In case the peak is low, a cut off value of 2000 was used for positive/negative judgement, and the test was repeated to confirm the consistency of the results.

### Specimen collection and testing

The research protocol, collection and use of clinical data were approved by the Research Ethics Board, Children’s Hospital of Hebei Province. Informed written consent was obtained from the parent of all participants. From March 2013 to February 2014, a total of 1699 nasopharyngeal swab specimens were collected from hospitalized patients diagnosed with CAP within 48 hours of admission after written informed consent was obtained from the parent of the participant. The diagnosis was based on CAP Diagnosis and Management Guidelines established by the Respiratory Society of Chinese Medical Association [17]. The nasopharyngeal specimen was collected into a transport tube containing 1 ml DMEM medium with 2% heat-inactivated fetal calf serum, 50 IU/ml of penicillin and 100 μg/ml of streptomycin (Gibco, Beijing, China). The sample was stored at 4˚C for the same day viral nucleic acid extraction. Two hundred μL of nasopharyngeal sample was spiked with 5 ng pcDNA3.1 (+) DNA and the rest 800 μL was stored at a -80°C freezer. The spiked sample was used for total viral nucleic acid (both DNA and RNA) extraction using the EasyPure Viral DNA/RNA Kit (QSJBio, Beijing, China) according to the manufacturer’s instructions. The final viral nucleic acid was eluted in 30 μL nuclease-free water. Afterwards, 3 μL of extracted nucleic acid was analyzed with the GeXP-based assay as described above.

### Statistical analysis

Positive percentages in male and female patients and positive percentages among different age groups were analyzed with chi-square test using the SPSS 13.0.1 statistics package (SPSS Inc., Chicago, USA). *p*<0.05 was considered statistically significant.

## Results

### Sensitivity and specificity of the GeXP-based assay

**Fig 1:**
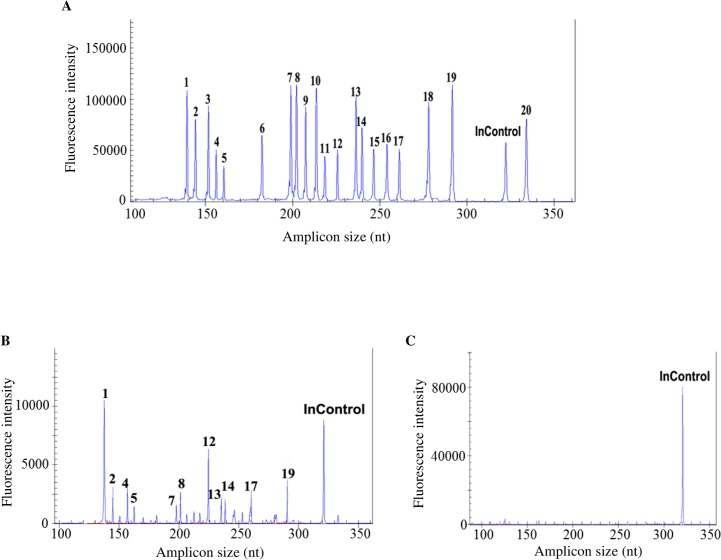
Sensitivity and specificity test of the GeXP assay. When the template pool was at the concentration of 10^4^ copy/μL, all 20 viruses in the target pool were successfully amplified (panel A), while at 10^3^ copy/μL, only FluA, ADV, HBoV, HRV, SARS, N2, PIV2, HMPV, swH1N1, H1 and HCoV-HKU1/OC43 were detected positive (panel B). Specificity analysis showed that all templates in the control pool were tested negative (panel C, 10^4^ copies of pcDNA3.1 (+) was used as control). InControl: internal control-pcDNA3.1 (+); 1: FluA; 2: ADV; 3: PIV1; 4: HBoV; 5: HRV; 6: HCoV NL63/229E; 7: SARS; 8: N2; 9: FluB; 10: PIV3; 11: H5N1; 12: PIV2; 13: HMPV; 14: swH1N1; 15: RSV; 16: SeH1N1; 17: H1; 18: H3; 19: HCoVOC43; 20: N1.

Sensitivity was determined using the serially diluted target pool, as shown in Fig 1A, at the concentration of 10^4^ copy/μL, all viruses in the target pool were detected as positive, while at 10^3^ copy/μL, FluA, ADV, HBoV, HRV, SARS, N2, PIV2, HMPV, swH1N1, H1 and HCoV-HKU1/OC43 were tested positive (Fig 1B). At 10^2^ copy/μL, only PIV2, ADV and HBoV were detected as positive while at 10^1^ copy/μL, none of the target viruses was amplified (both data not shown). Specificity test showed that all templates from the control pool were tested negative (Fig 1C). Those tiny peaks shown in panel C were further confirmed to be background (see S1 Fig).

### Nasopharyngeal specimens testing

Nasopharyngeal specimens collected from 1699 hospitalized children (male: 878; female: 821) aged from 5 days to 13 years were tested with the GeXP assay. Demographics of all patients were listed in Table 2. A total of 1096 samples (64.51%, 1096/1699) were positive for at least one virus, among which, coinfection was observed in 165 cases. Viruses discovered in the samples included RSV, PIV3, HRV, ADV, HBoV, PIV1, HMPV, FluA, HCoV, FluB, N1 and N2, among which RSV was the most detected followed by PIV3, HRV, ADV, and HBoV (Table 3).

**Table 2:** Demographics of pediatric patients with CAP.

Male	Female	Total	Interquartile range of age (months)
878	821	1699	3–13

**Table 3:** Top 5 viruses detected in nasopharyngeal samples isolated from patients with CAP.

Virus	Numbers of positive samples
RSV	391
PIV3	246
HRV	234
ADV	100
HBoV	63

### Virus prevalence in different groups of patients

The positive rates in samples collected from males and females were 66.10% and 62.8%, respectively, the difference was not statistically significant (*p* = 0.16). Sample positivity was further compared among 4 different age groups, i.e., <1 year, 1–3 years, 3–5 years and ˃5 years. As shown in Table 4, positive rates for the groups of <1 year and 1–3 years are significantly higher than those in groups of 3–5 years and ˃5 years, respectively.

**Table 4:** Virus prevalence in different age of groups.

Group	Total sample number	Positive sample number	Positive percentage
< 1 year	601	442	73.54%*
1–3 years	675	476	70.52%*
3–5 years	351	149	42.45%
> 5 years	72	29	40.28%

* *p<*0.01 compared with 3–5 years and > 5 years groups, respectively.

### Virus prevalence in different seasons

Seasonal distribution analysis revealed that RSV was the most predominant in autumn and winter, while in spring and summer PIV3 and RSV were the most frequently identified with similar positive percentages (Table 5).

**Table 5:** Top 5 virus detected in each season.

Sequence	Spr (% positive)	Sum (% positive)	Aut (% positive)	Win (% positive)
1	PIV3 (30.3%)	RSV (25.7%)	RSV (33.7%)	RSV (37.0%)
2	RSV (28.2%)	PIV3 (25.7%)	PIV3 (25.5%)	HRV (17.7%)
3	HRV (18.7%)	HRV (22.4%)	HRV (20.7%)	PIV3 (8.5%)
4	ADV (10.6%)	ADV (10.9%)	ADV (5.8%)	ADV (7.4%)
5	HBoV (5.3%)	HBoV (6.0%)	HBoV (5.4%)	HMPV (6.5%)

Spr: Spring; Sum: Summer; Aut: Autumn; Win: Winter.

## Discussion

Nucleic acid amplification methods such as PCR and RT-PCR have increasingly been explored for identification of pathogens including virus detection in infectious respiratory diseases [9, 16, 18]. So far, only a few reports have described the use of these techniques for pathogen detection in children with CAP [7, 11, 12]. Determination of viral etiology for pediatric CAP in larger clinical series has not been reported. In the present study, we applied multiplex RT-PCR together with automated capillary electrophoresis, namely the GeXP-based assay, to detect virus in 1699 nasopharyngeal specimens from hospitalized children with CAP. We showed that the GeXP-based assay had high sensitivity and specificity for simultaneous detection of multiple viruses, and about 65% of cases tested were positive for virus. We randomly chose 120 samples that had been tested by the GeXP-based assay, redo the mono-RT-PCR and found 97.5% diagnosis agreement between these 2 methods (data not shown, and mono-RT-PCR was run as the multiplex reactions but used only the chimeric and universal primers for the single target virus that was tested positive in the sample). Further analysis revealed that patients younger than 3 years had a significantly higher virus infection rate (positive rate) than those older than 3 years. Seasonal distribution analysis saw RSV as the most predominant in autumn and winter, while in spring and summer PIV3 and RSV were the most frequently identified with similar positive percentages.

Only a few reports have thus far described different assays for virus detection in pediatric CAP patients [7, 11, 12, 19]. Chen et al measured antibodies in 1204 serum samples collected from CAP children and found only about 15% were positive for virus [19], in sharp contrast to our findings and others’ [7, 11, 12]. This discrepancy is likely attributed to 1) that the antibody assay targeted only a few types of viruses, i.e., influenza A and B, parainfluenza 1, 2 and 3 and RSV [19]; and 2) that the antibody assay is less sensitive than PCR [7, 16]. Juven et al explored several methods, i.e., cell culture, viral antigen detection, antibody assay and RT-PCR to determine the etiology of CAP in 254 hospitalized children, and they discovered that 62% of the patients had viral infection with RSV (29%) and HRV (24%) being the most commonly detected [7]. Cantais tested 9 types of viruses, i.e., RSV, ADV, HMPV, HBoV, coronavirus, FluA, FluB, HRV and PIVs using the biplex commercially-available RT-PCR Kit in 85 children with CAP, and revealed that the viral infection was 62.4% [11]. Rhedin et al employed real time PCR to detect virus in nasopharyngeal aspirates from children aged ≤5 years with CAP, and reported that viruses were detected in 81% of the cases [12]. These data together with ours suggest that majority of pediatric CAP cases have virus infection, especially in younger patients.

To our knowledge, this study is the first to use the GeXP-based assay for virus detection in pediatric CAP patients. The assay covered a broad range of virus targets and a large size of samples were tested. Due to its automation and multiplex procedure, the GeXP-based assay is less laborious than mono-PCR. It also saves samples and avoids well-to-well and pipetting errors. In the present study, plasmid DNA was used as the internal control, which monitors only PCR but not the reverse transcription step. Interestingly, in a few published papers describing the GeXP-based multiplex assays internal controls were not employed [13–15]. We showed that genomic RNA from all RNA viruses in the target panel was successfully amplified by the GeXP-based assay (Fig 1A), indicating that reverse transcription is not a problem. However, we felt an RNA template should be used as the internal control in future work. Another limitation of this assay is that highly specialized laboratory equipment is needed, namely an automated capillary electrophoresis system. Many clinical laboratories do not have this equipment, and outsourcing the fragment analysis would greatly increase the turnaround time.

## Supporting Information

S1 Data Accessible data of all participants. (XLS)

S1 Fig Further specificity analysis to identify those tiny peaks shown in Fig 1C. Control template pool containing 1 ng nucleic acid of each control pathogen and 10^3^copies of internal control was used and the reaction was repeated. As shown in this scaled-down plot, all those tiny peaks were identified as background products. Similar results were observed in 3 independent reactions.
(TIF)
