# Supplementary figures and images for: A GeXP-Based Assay for Simultaneous Detection of Multiple Viruses in Hospitalized Children with Community Acquired Pneumonia
Source: PLoS One. 2016 Sep 14;11(9):e0162411. doi: 10.1371/journal.pone.0162411 (PMC5023126; doi:10.1371/journal.pone.0162411)

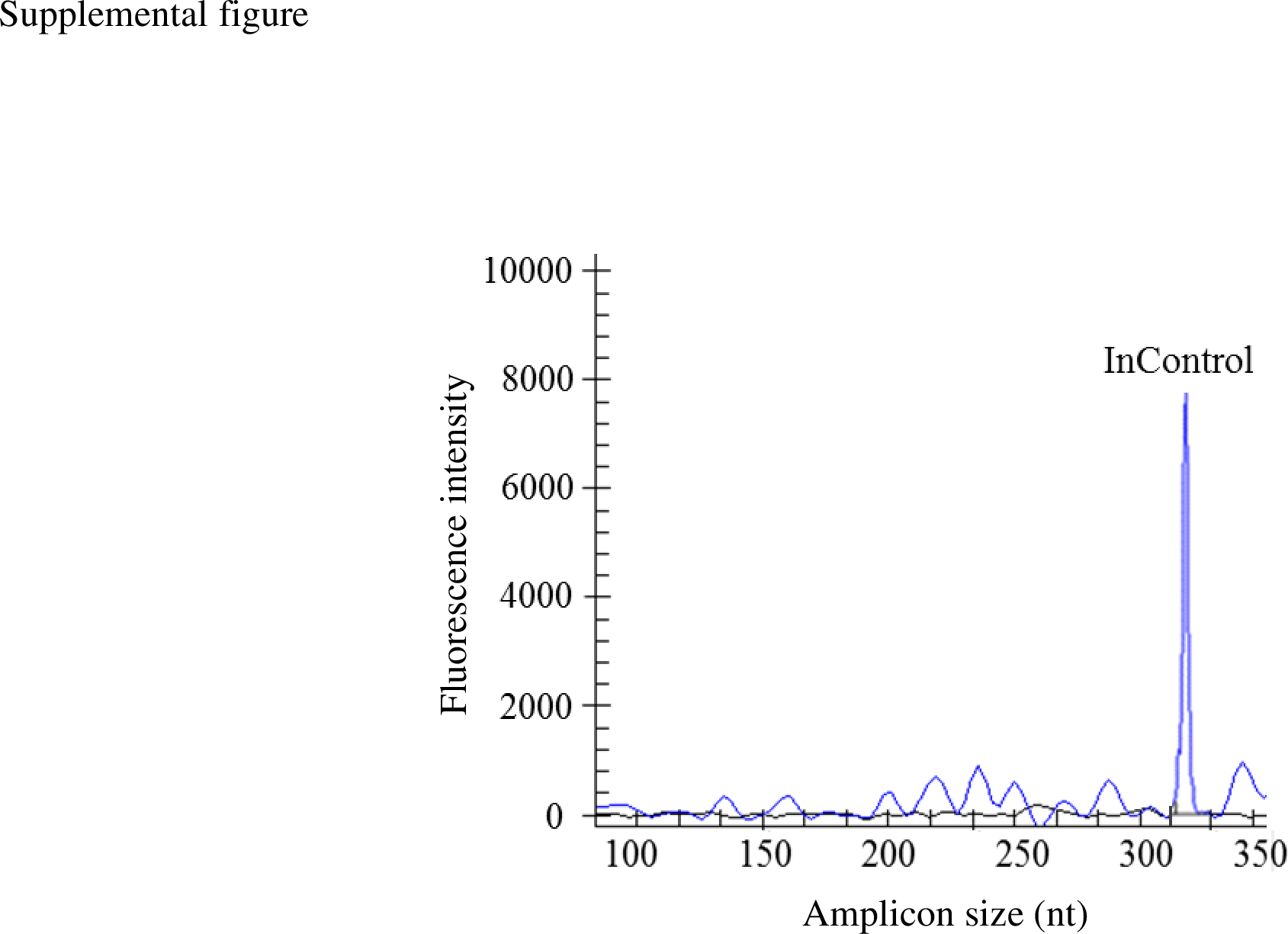

Supplement: S1 Fig — Control template pool containing 1 ng nucleic acid of each control pathogen and 103copies of internal control was used and the reaction was repeated. As shown in this scaled-down plot, all those tiny peaks were identified as background products. Similar results were observed in 3 independent reactions. (TIF) [file pone.0162411.s002.tif]
